# Allogeneic transplantation of mobilized dental pulp stem cells with the mismatched dog leukocyte antigen type is safe and efficacious for total pulp regeneration

**DOI:** 10.1186/s13287-018-0855-8

**Published:** 2018-04-27

**Authors:** Koichiro Iohara, Shinji Utsunomiya, Sakae Kohara, Misako Nakashima

**Affiliations:** 1 Department of Stem Cell Biology and Regenerative Medicine, National Center for Geriatrics and Gerontology, Research Institute, 7-430 Morioka, Obu, Aichi, 474-8511 Japan; 2 Drug Safety Research Laboratories, Shin Nippon Biomedical Laboratories Ltd, Kagoshima, Japan; 3 Preclinical Research Support Division, Shin Nippon Biomedical Laboratories Ltd, Kainan, Japan

**Keywords:** Allogeneic cell transplantation, Pulp regeneration, Mobilized dental pulp stem cells, Granulocyte-colony stimulating factor, Pulpectomy, Dog leukocyte antigen, Dual transplantation

## Abstract

**Background:**

We recently demonstrated that autologous transplantation of mobilized dental pulp stem cells (MDPSCs) was a safe and efficacious potential therapy for total pulp regeneration in a clinical study. The autologous MDPSCs, however, have some limitations to overcome, such as limited availability of discarded teeth from older patients. In the present study, we investigated whether MDPSCs can be used for allogeneic applications to expand their therapeutic use.

**Methods:**

Analysis of dog leukocyte antigen (DLA) was performed using polymerase chain reaction from blood. Canine allogeneic MDPSCs with the matched and mismatched DLA were transplanted with granulocyte-colony stimulating factor in collagen into pulpectomized teeth respectively (*n* = 7, each). Results were evaluated by hematoxylin and eosin staining, Masson trichrome staining, PGP9.5 immunostaining, and BS-1 lectin immunostaining performed 12 weeks after transplantation. The MDPSCs of the same DLA used in the first transplantation were further transplanted into another pulpectomized tooth and evaluated 12 weeks after transplantation.

**Results:**

There was no evidence of toxicity or adverse events of the allogeneic transplantation of the MDPSCs with the mismatched DLA. No adverse event of dual transplantation of the MDPSCs with the matched and mismatched DLA was observed. Regenerated pulp tissues including neovascularization and neuronal extension were quantitatively and qualitatively similar at 12 weeks in both matched and mismatched DLA transplants. Regenerated pulp tissue was similarly observed in the dual transplantation as in the single transplantation of MDPSCs both with the matched and mismatched DLA.

**Conclusions:**

Dual allogeneic transplantation of MDPSCs with the mismatched DLA is a safe and efficacious method for total pulp regeneration.

**Electronic supplementary material:**

The online version of this article (10.1186/s13287-018-0855-8) contains supplementary material, which is available to authorized users.

## Background

Pulp/dentin complex in teeth has a critical function in the maintenance of tooth homeostasis, and viable pulp is essential for the longevity of the tooth [1]. The ultimate goal for regenerative endodontics is to replace or restore the impaired or damaged tissues with viable pulp tissue in the case of pulpitis or apical periodontitis, leading to the reestablishment of the physiologic, structural, and mechanical integrity of the native dentin/pulp complex, including function of pulp immunity, dentin formation, pulp innervation, and vascular perfusion [1–4]. Stem cell therapy has been suggested as an effective regenerative technique for pulpitis and apical periodontitis. Autologous transplantation of dental pulp stem cell (DPSC) subsets, dental pulp-derived CD31^−^ side population (SP) cells, or CD105^+^ cells with SDF1 in orthotopic sites demonstrated complete pulp regeneration [5, 6]. Subsequently, colony-derived DPSCs with platelet-rich plasma/fibrin (PRP/PRF) showed similar successful results [7]. The safety and efficacy of autologous DPSC therapy were demonstrated in the preclinical study harnessing DPSCs mobilized (MDPSCs) with granulocyte-colony stimulating factor (G-CSF) harvested in good manufacturing practice conditions [8]. Furthermore, a recent clinical study suggested autologous MDPSC transplantation may be safe and efficacious for pulp regeneration in humans [9]. The autologous DPSCs, however, have certain limitations to overcome, such as limited availability of human discarded teeth and the high cost of the safety and quality control tests of individual cell products before transplantation. The further potential disadvantages of the autologous mesenchymal stem cells (MSCs) are their decreased biological activity from older patients and altered intrinsic stem cell properties from patients with some systemic diseases including diabetes and rheumatoid arthritis [10]. Transplantation of autologous mobilized adipose-derived or bone marrow-derived MSCs resulted in lower volume of regenerated pulp tissue, less angiogenesis, and reinnervation compared with MDPSCs [11]. Furthermore, regenerated pulp tissues in adipose and bone marrow MSC transplants were more mineralized compared with MDPSC transplant, suggesting pulp MSCs were an optimal cell source for pulp regeneration. Thus, banked allogeneic DPSCs would be highly advantageous to save time and costs and to confirm high quality [12].

The use of allogeneic MSCs permits low immunogenicity with immunomodulatory and immunosuppressive properties. It is well known that MSCs have low immunogenicity due to no expression of class II major histocompatibility complex (MHC-II) proteins, and low or modest expression of MHC-I proteins and costimulatory molecules such as CD40, CD80, and CD86 on their cell surface [13, 14]. Therefore, MSCs are unable to provoke a cytotoxic effect by allogeneic immune cells [15], and MSCs from MHC-mismatched donors may also be used for cell therapy [16]. Many studies recently focused on mechanisms of immunomodulation and immunosuppression of MSCs, especially in reducing inflammation, escaping from immune cell response, and modulating T-cell proliferation. MSCs can interfere with different pathways of the immune responses by means of direct cell-to-cell interactions and secretion of soluble factors such as transforming growth factor (TGF)-β, hepatocyte growth factor (HGF), prostaglandin E2 (PGE2), nitric oxide (NO), indoleamine-2,3 dioxygenase (IDO), tumor necrosis factor (TNF)-α stimulated gene-protein 6, interleukin (IL)-6, IL-10, semaphorin-3A, galectin (Gal)-1, and Gal-9 [17–20]. MSCs also possess the ability to generate regulatory T cells (Tregs) which suppress other immune cells [21, 22]. The whole range of mechanisms of immunomodulation and immunosuppression mediated by MSCs remains incompletely understood. The immunosuppressive and immunomodulatory responses are, however, properties shared by MSCs from a variety of adult and fetal tissues including dental pulp [18, 23].

A number of animal experiments have demonstrated that allogeneic MSCs improve acute myocardial infarction, chronic spinal cord injury, ischemic stroke, fracture healing, and osteoarthritis by local injection or intravenous infusion [24–28]. No adverse effects have been noticed in 291 equine recipients over a period of up to 1 year after intravenous injection of allogeneic peripheral blood-derived MSCs [29]. On the other hand, MHC class I mismatched MSCs induced CD8^+VE^, CD16^+VE^, and CD8^+VE^/CD16^+VE^ lymphocyte subpopulations, dependent on the dose of administered MSCs in intracranial injection and the degree of antigenic mismatch between donor and recipient [30]. Allogeneic bone marrow MSC transplantation into infarcted rat myocardium improved ventricular function for 3 months and a delayed immune rejection/response has been reported within 5 months due to the shift from a hypoimmunogenic to an immunogenic state of the transplanted MSCs upon differentiation [31]. Thus, consistent results have not yet been obtained on the therapeutic effects of allogeneic MSCs, depending on routes, timing duration, and dosage of MSC administration in vivo [10].

It is desirable for MSC transplantation into the root canal of the tooth to be repeated for pulp regeneration in patients with multiple caries. Repeated injection of allogeneic adipose tissue-derived MSCs (AT-MSCs) or bone marrow-derived MSCs (BM-MSCs) demonstrated a lack of adverse effects. However, repeated BM-MSC injections resulted in an increase in blood CD8^+^ T-cell numbers and splenic regulatory T-cell numbers compared with AT-MSCs in healthy horses, indicating a mild alloantigen-directed cytotoxic response [32]. Repeated intravenous injection of allogeneic porcine bone marrow MSCs or human umbilical cord blood-derived MSCs also induced no immunological alterations including T-cell proliferation, high levels of IFN-γ, TNF-α, and human IgG and no adverse events due to low immunogenicity [33, 34]. Dual allogeneic MSC treatment by transepicardial injection in the acute and the subacute period after myocardial infarction improved ventricular function with increased myocardial mass and anteriolar density more than single MSC treatment in rats [35].

There are no reports on MHC-mismatched allogeneic transplantation and on dual allogeneic MSC transplantation in pulp regenerative therapy. Thus, there is a need to address challenges to allogeneic MDPSC cell therapy for total pulp regeneration. The aim of this investigation is to assess the safety and efficacy of allogeneic transplantation of canine MDPSCs into the pulpectomized tooth. The dual consecutive transplantation was further evaluated for safety and efficacy compared with the single transplantation.

## Methods

### Dog leukocyte antigen genotyping and matching analysis

We used beagle dogs (Kitayama Labes, Iwakuni and Ina, Japan) owned by Shin Nippon Kagaku Biomedical Laboratories Ltd (*n* = 26). Total genomic DNA was extracted from whole blood of dogs by NuclesaseMag®96 Blood (Marcherey-Nagel, Düren, Germany) according to the protocol. All of the dogs in the investigation were not in a sibling relationship. Genotyping was performed by direct sequencing and sequencing of the polymerase chain reaction (PCR) product. PCR was performed using primers *DLA-88 exon 1–3* (1100 bp), *DLA-DQA exon 2* (300 bp),* DLA-DQB exon 2* (350 bp), and *DLA-DRB exon 2* (350 bp) [36, 37] (Table 1) with KOD Fx (TOYOBO Co., Ltd, Osaka, Japan) in a GeneAmp PCR system 9700 (Thermo Fisher Scientific K.K., Yokohama, Japan). PCR products were subcloned into a ZeroBlunt®TOPO PCR Cloning Kit (Thermo Fisher Scientific K.K.). Sequencing was carried out using a ABI PRISM BigDye Terminator v3.0 Ready Reaction Cycle Sequencing Kit (Thermo Fisher Scientific K.K.) with an ABI PRISM 3730 DNA Analyzer (Thermo Fisher Scientific K.K.), and the raw data were analyzed by Sequencer Ver 4.8 (Gene Codes Corp., Ann Arbor, MI, USA). The allele names were determined according to the universal nomenclature found in the Immuno Polymorphism Database (EMBL-EBI, Cambridge, UK).

**Table 1:** Primers of polymerase chain reaction for dog leukocyte antigen (DLA) genotyping

Gene		5′–3′ DNA sequence
*DLA-88 exon 1–3*	Forward	AGTCCAGCGGCGACGGCCAGTGTCCCCGGA
	Reverse	AGCCCTCCCTAGTGGAGGCGAGATCGGGGA
*DLA-DQA exon 2*	Forward	TAAGGTTCTTTTCTCCCTCT
	Reverse	GG AC AG ATT C AGT G AAG AG A
*DLA-DQB exon 2*	Forward	CTCACTGGCCCGGCTGTCTC
	Reverse	GGTGCGCTCACCTCGCCGCT
*DLA-DRB exon 2*	Forward	GATCCCCCCGTCCCCACAG
	Reverse	TGTGTCACACACCTCAGCACCA

### Cell isolation and culture

Upper left lateral incisors were freshly extracted from each beagle dog at 8 months of age. After making a longitudinal cut, they were transported by air within 30 h to the National Center for Geriatrics and Gerontology (NCGG) from Shin Nippon Biomedical Laboratories Ltd, Drug Safety Research Laboratories (Kagoshima, Japan), and RaQualia Pharma Inc. (Rental Laboratories of NCGG) in Hank’s balanced salt solution (Invitrogen, Carlsbad, CA, USA) with 2.5 mg/ml amphotericin B (Bristol-Myers Squibb, Tokyo, Japan) and 0.3% gentamicin (Nitten, Nagoya, Japan). Mobilized dental pulp stem cells (MDPSCs) were isolated using the similar procedure used for a previous autologous preclinical study [8]. In brief, dental pulp tissues isolated from the teeth were enzymatically digested in 0.04 mg/ml Liberase (Roche, Mannheim, Germany) for 30 min at 37 °C. The isolated pulp cells were plated at 2 × 10^4^ cells on T-25 (Asahi Technoglass, Funabashi, Japan) in Dulbecco’s Modified Eagle’s Medium (DMEM) (Sigma, St. Louis, MO, USA) supplemented with 10% autologous canine serum, 2.5 mg/ml amphotericin B, and 0.3% gentamicin. They were detached by incubation with TrypLE™ Select (Invitrogen) prior to 70% confluence. The colony-formed DPSCs were further isolated by G-CSF-induced stem cell mobilization method with G-CSF (Neutrogin; Chugai Pharmaceutical Co., Ltd, Tokyo, Japan) at 100 ng/ml, with 2 × 10^4^ cells/100 μl on the Transwell (Corning, Lowell, MA, USA), and inserted into 24-well tissue culture plates for 48-h incubation [38]. The isolated MDPSCs were detached by incubation with TrypLE™ Select at 60–70% confluence and subcultured at a 1:3 dilution into cell culture flasks (25 cm^2^ and further 75 cm^2^) (Asahi Technoglass) in DMEM supplemented with 10% autologous canine serum without antibiotics. The cells were cryopreserved at a cell concentration of 1 × 10^6^ cells/ml in an extracellular cryoprotectant (CP-1; Kyokuto Pharmaceutical Industrial Co., Ltd, Tokyo, Japan) at the 7th passage of culture.

### Characterization of mobilized dental pulp stem cells

The quality of cryopreserved MDPSCs was confirmed by cell viability and proliferation abilities at the 7th passage of culture. In brief, the cells stained with trypan blue (Sigma-Aldrich, St. Louis, MO, USA) were counted following thawing for the cell viability test, further plated at 2.0 × 10^5^ cells in 10-cm dishes (Corning, NY, USA), and proliferation rates were calculated at 48 h as the doubling time.

To further characterize the immunomodulation ability of MDPSCs, mobilized adipose-derived stem cells (MADSCs) were isolated from the abdominal subcutaneous adipose tissue [11] from the same individual dog similarly to MDPSCs as already described for comparison with MDPSCs. Total RNA was extracted with TRIzol (Life Technologies). First-strand cDNA syntheses were performed on the total RNA of these cells by reverse transcription using ReverTra Ace-a (Toyobo, Tokyo, Japan) after DNase I treatment (Roche Diagnostics) at 37 °C for 20 min. Real-time RT-PCR was performed using primers for the immunomodulatory factors (Table 2) *prostaglandin E synthase* (*PTGES*), *cyclooxygenase-2* (*COX-2*), *IL-6*, *TGF-β* and *indoleamine 2, 3-dioxygenase-1* (*IDO-1*) labeled with AmpliTaq Gold master mix (Thermo Fisher Scientific) in an Applied Biosystems® 7500 Real-Time PCR (Life Technologies). After normalizing with *β-actin*, the mRNA level of each immunomodulatory factor in MDPSCs was compared with that in MADSCs derived from the same individual dog (*n* = 3).

**Table 2:** Canine primers for real-time reverse transcription-polymerase chain reaction for immunomodulatory factors

Gene		5′–3′ DNA sequence	Accession number	Product (base pairs)
*PTGES*	Forward	GCCGCTGTGACTGTACC	NM_001122854	190
	Reverse	TGGTCCAATCAGCCACTTC		
*COX-2*	Forward	GTTCATTCCTGATCCCCAAG	NM_001003354	186
	Reverse	TTGAAAAGGCGCAGTTTATG		
*IL-6*	Forward	TCCAGAACAACTATGAGGGTGA	NM_001003301.1	l00
	Reverse	TCCTGATTCTTTACCTTGCTCTT		
*TGF-β*	Forward	CTGGAGTCGTGAGGCAGTG	NM 0010033309.1	96
	Reverse	GCAGTGTGTTATCTTTGCTGTCA		
*IDO-1*	Forward	GGAAAGGCAACTCCAAACTG	XM_532793	124
	Reverse	CCCAGCAGAATGTCAAAGC		
*β-actin*	Forward	AAGTACCCCATTGAGCACGG	Z70044	257
	Reverse	ATCACGATGCCAGTGGTGCG		

PTGES prostaglandin E synthase, COX-2 cyclooxygenase-2, IL interleukin, TGF transforming growth factor, IDO-1 indoleamine 2,3-dioxygenase 1.

### Evaluation of safety of first and second allogeneic transplantation for pulp regeneration

**Fig. 1:**
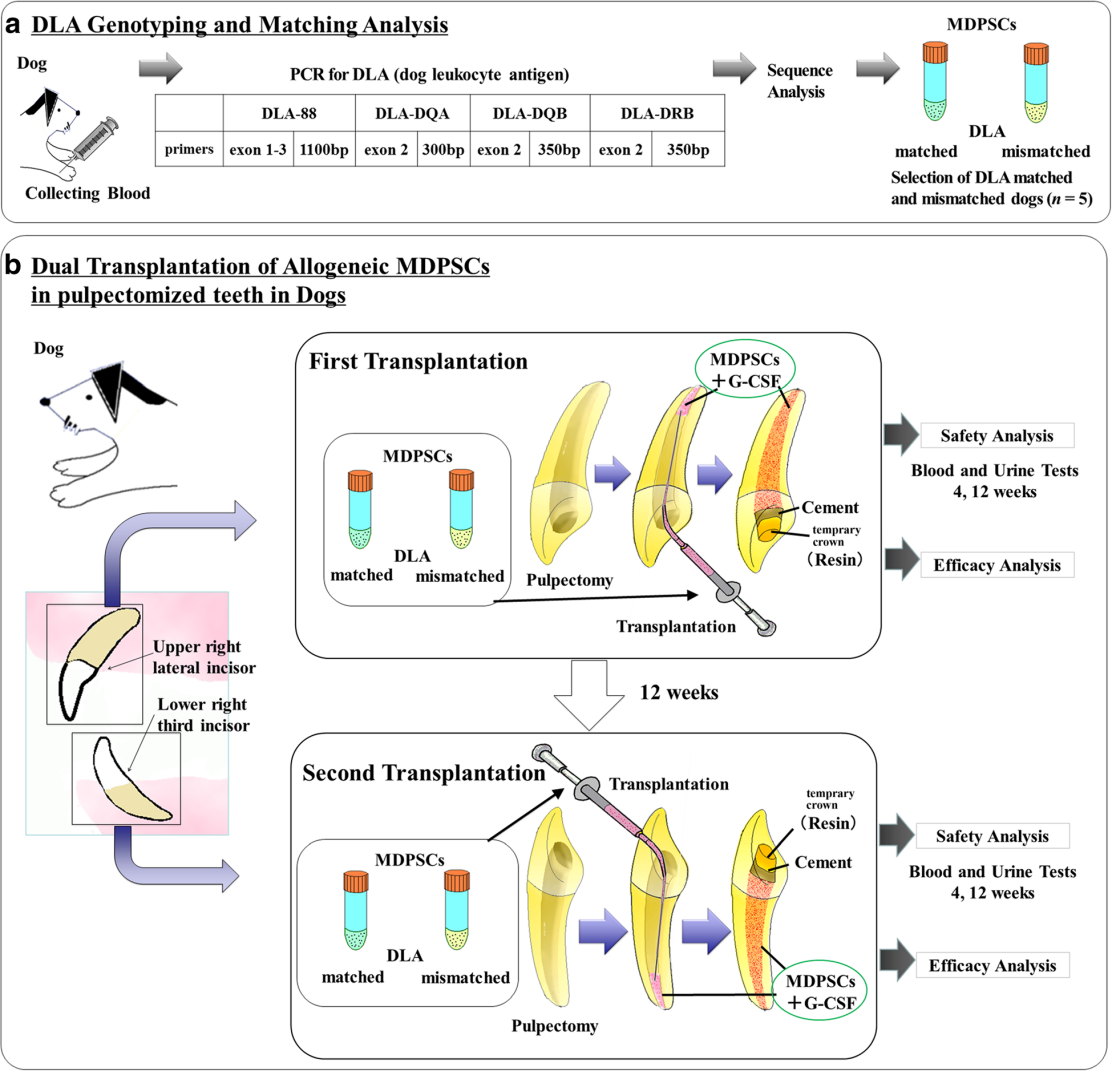
Schematic diagram of allogeneic pulp regeneration after transplantation of mobilized dental pulp stem cells (MDPSCs) in a canine pulpectomy model in teeth. **a** Analysis of allele profiles. Dog leukocyte antigen (DLA) genotyping and matching analysis from whole blood of dogs. **b** Dual transplantation of allogeneic MDPSCs in pulpectomized teeth in dogs. First transplantation: DLA matched and mismatched MDPSCs transplanted in the upper right lateral incisors. Safety and efficacy analyses performed at 4 and 12 weeks and at 12 weeks, respectively. Second transplantation: the same matched and mismatched MDPSCs as the first transplantation transplanted in the lower right third incisors in the same dogs. At 12 weeks after second transplantation, histological examination of all organs including the transplanted teeth and surrounding periodontal tissue performed for the safety test in addition to the same safety and efficacy test as the first transplantation. PCR polymerase chain reaction, G-CSF granulocyte colony-stimulating factor, temprary crown

The cryopreserved MDPSCs were transported by air to the operating room of the animal facility in Shin Nippon Biomedical Laboratories Ltd under strict temperature control. One day before transplantation, the root canal was open to the apex with a #25 K file after pulpectomy and shaped to 0.55 mm in width, 0.5 mm from the apex in the upper right lateral incisors in 14 dogs. For the first allogeneic transplantation, the dog leukocyte antigen (DLA) matched and mismatched MDPSCs (*n* = 5, respectively) were transplanted into the root canal, 5 × 10^5^ cells together with 20 μl of collagen scaffold (atelocollagen implant; Koken, Tokyo, Japan) and 150 ng of G-CSF (Neutrogin), respectively (Fig. 1). For toxicology assessment, clinical signs of dogs were observed and their food consumption was measured daily, and their weights were recorded weekly. Urine chemistry examinations by Clinitek AtlasXL (Sparton Medical Systems, Strongsville, OH, USA) were performed at 4 and 12 weeks, and blood tests by ADIVIA 120 (Siemens Healthcare Diagnostics Manufacturing Ltd, Erlangen, Germany) and blood chemistry examinations by JCA-BM6070 (Japan Electron Optical Laboratory, Tokyo, Japan) were performed at 4 and 12 weeks after transplantation. Blood tests demonstrate the red blood cell count (RBC) and hematocrit (Ht) for homeostasis of blood cells, the platelet count (Plt) for inflammation, and the white blood cell count (WBC) for inflammation and infection. Blood chemistry examinations demonstrate aspartate transaminase (AST) and alanine transaminase (ALT) for abnormality of the liver, albumin and globulin for protein metabolism, total cholesterol (T-cho) for the lipid profile, and glucose for abnormality of hormone. After extraction of the upper right lateral incisors at 12 weeks, the second allogeneic transplantation of the same matched and mismatched MDPSCs as the first transplantation was performed in pulpectomized lower right third incisors in the same dogs, respectively (*n* = 5). The safety tests were further performed at 4 and 12 weeks. The transplanted teeth were extracted at 24 weeks followed by euthanization. All organs were weighed and macroscopically examined. Furthermore, histopathological examination of all organs and tissues including the transplanted teeth with surrounding periodontal tissue were also performed in the paraffin sections stained with hematoxylin and eosin (HE).

### Efficacy of first and second allogeneic transplantation for pulp regeneration

Morphological examination of the regenerated tissue was performed in the paraffin sections (5 μm in thickness) of the teeth. The relative amounts of regenerated tissue were measured in the sections of the first transplants of matched (*n* = 5) and mismatched MDPSCs (*n* = 5), and the second transplants of matched (*n* = 4) and mismatched MDPSCs (*n* = 5). On-screen image outlines of regenerated tissue in the root canals were traced by capturing images of the histological preparations on a binocular microscope (M 205 FA; Leica) and the surface area of these outlines was determined using Leica Application Suite software (version 3.4.1; Leica).

For neovascularization and innervations analyses, 5-μm-thick paraffin sections were deparaffinized and stained with Fluorescein Griffonia (Bandeiraea) Simplicifolia Lectin 1/fluorescein-galanthus nivalis (snowdrop) lectin (BS-1 lectin) (20 μg/ml; Vector laboratories, Inc., Youngstown, OH, USA) and anti-PGP9.5 (Ultra Clone) (1:10,000), respectively, as described previously [6]. The ratios of the BS-1 lectin-positive newly formed capillaries were calculated respectively using a Dynamic cell count BZ-HIC (KEYENCE, Osaka, Japan) in the first transplants of matched (*n* = 4) and mismatched MDPSCs (*n* = 4), and in the second transplants of matched (*n* = 5) and mismatched (*n* = 5) MDPSCs.

For quantitative analysis of matrix formation, the sections from each four teeth at 12 weeks after the first and second transplantation of matched and mismatched MDPSCs were stained with Masson trichrome staining (Muto Pure Chemicals Co., Ltd, Tokyo, Japan). The relative amounts of matrix formation area were measured in the sections of the first transplants of matched (*n* = 5) and mismatched MDPSCs (*n* = 5), and the second transplants of matched (*n* = 4) and mismatched MDPSCs (*n* = 3). On-screen image outlines were traced by capturing images of the histological preparations on a binocular microscope (M 205 FA; Leica). The positive area was quantitatively analyzed using Leica Application Suite software (version 3.4.1; Leica).

### Statistical analyses

Data are reported as mean ± SD. *P* values were calculated using Tukey’s multiple comparison test method in SPSS 21.0 (IBM, Armonk, NY, USA).

## Results

### DLA analysis

DLA genotyping and matching analyses in 26 dogs demonstrated a four homozygous allele profile (nine dogs), a three homozygous and one heterozygous allele profile (three dogs), a two homozygous and two heterozygous allele profile (four dogs), a one homozygous and three heterozygous allele profile (one dog), and a four heterozygous allele profile (nine dogs). In the four homozygous allele profile group, eight dogs had eight completely matched alleles (Group A) out of nine dogs. In the two homozygous and two heterozygous allele profile group, four dogs had seven matched alleles. In the four heterozygous haplotype group, four dogs had seven matched alleles (Group B) out of nine dogs (Table 3). We selected five identical and almost identical donors of the allele profiles (four dogs from Group A, one dog from Group B) and five nonidentical donors with at least four mismatched alleles for allogeneic transplantation (Table 4).

**Table 3:** Dog leukocyte antigen (DLA) analysis of the 26 individual dogs

Group	Animal number	Gender	DLA-88		DLA-DQA		DLA-DQB		DLA-DRB		Haplotype
			exon 1–3 (ll00 bp)		exon 2 (300 bp)		exon 2 (350 bp)		exon 2 (350 bp)		
A	13MW 302	Male	*50201	*50201	*00101	*00101	*00201	*00201	*00102	*00102	4 homo
A	13MW 320	Male	*50201	*50201	*00101	*00101	*00201	*00201	*00102	*00102	4 homo
A	13MW1036	Male	*50201	*50201	*00101	*00101	*00201	*00201	*00102	*00102	4 homo
A	BMW 1052	Male	*50201	*50201	*00101	*00101	*00201	*00201	*00102	*00102	4 homo
A	13FW 191	Female	*50201	*50201	*00101	*00101	*00201	*00201	*00102	*00102	4 homo
A	13FW283	Female	*50201	*50201	*00101	*00101	*00201	*00201	*00102	*00102	4 homo
A	13FW 956	Female	*50201	*50201	*00101	*00101	*00201	*00201	*00102	*00102	4 homo
A	13FW 976	Female	*50201	*50201	*00101	*00101	*00201	*00201	*00102	*00102	4 homo
	13MW1149	Male	*11	*11	*00101	*00101	*00201	*00201	*00101	*00101	4 homo
	BMW 273	Male	*02501	*16a	*005011	*005011	*00701	*00701	*02801	*02801	3 homo 1 hetero
	13FW 1026	Female	*50201	*50801	*00101	*00101	*00201	*00201	*00102	*00102	3 homo 1 hetero
	13FW300	Female	*50201	*50201	*00101	*00101	*00201	*00201	*00101	*00102	3 homo 1 hetero
	BMW 318	Male	*50201	*50801	*00101	*00101	*00201	*00201	*00101	*00102	2 homo 2 hetero
	13FW 992	Female	*50201	*50801	*00101	*00101	*00201	*00201	*00101	*00102	2 homo 2 hetero
	13FW 299	Female	*50201	*11	*00101	*00101	*00201	*00201	*00101	*00102	2 homo 2 hetero
	13FW1031	Female	*50201	*11	*00101	*00101	*00201	*00201	*00101	*00102	2 homo 2 hetero
	13FW281	Female	*50201	*50201	*00101a	*00301	*00201	*00401	*00102	*00801	1 homo 3 hetero
B	BMW 223	Male	*01201	*50201	*00101	*00901	*00101	*00201	*00102	*01501	4 hetero
B	13MW1133	Male	*01201	*50201	*00101	*00901	*00101	*00201	*00102	*01501	4 hetero
B	BMW 1029	Male	*01201	*50801	*00101	*00901	*00101	*00201	*00102	*01501	4 hetero
B	13FW 926	Female	*01201	*21	*00101	*00901	*00101	*00201	*00102	*01501	4 hetero
	13MW1135	Male	*01201	*11	*00101	*00901	*00101	*00201	*00101	*01501	4 hetero
	BMW 1086	Male	*50201	*11	*00101	*00301	*00201	*00401	*00101	*00801	4 hetero
	13FW1019	Female	*50201	*50801	*00101	*00901	*00101	*00201	*00102	*00201	4 hetero
	13FW 995	Female	*50801	*11	*00101	*00201	*00201	*01303	*00101	*00102	4 hetero
	13MW1122	Male	*02501	*50201	*00101	*005011	*00201	*00701	*00101	*02801	4 hetero

*bp* base pairs, *homo* homozygous, *hetero* heterozygous, * indicate alleles, “a” indicates the closest matching allele

**Table 4:** Dog leukocyte antigen (DLA) matched and mismatched MDPSC transplantation for safety and efficacy tests

Recipient dogs			Donor dogs of transplanted MDPSCs		Matched/mismatched
Individual number	DLA type	Gender	Individual number	DLA type	
13MW 302	4 homo	Male	BMW 320	4 homo	Complete matched
13FW 191	4 homo	Female	13FW283	4 homo	Complete matched
13FW 283	4 homo	Female	13FW 191	4 homo	Complete matched
13FW 956	4 homo	Female	13FW 976	4 homo	Complete matched
13MW 1029	4 hetero	Male	BMW 1133	4 hetero	Matched
13FW300	3 homo 1 hetero	Female	BMW 273	3 homo 1 hetero	Mismatched
13FW 299	2 homo 2 hetero	Female	BMW 223	4 hetero	Mismatched
13FW1031	2 homo 2 hetero	Female	13FW 1122	4 hetero	Mismatched
13FW926	4 hetero	Female	13FW 1031	2 homo 2 hetero	Mismatched
13MW 1122	4 hetero	Male	BMW 1149	4 homo	Mismatched

*MPDSC* mobilized dental pulp stem cell, *homo* homozygous, *hetero* heterozygous

### The isolated canine MDPSCs

The isolated and cryopreserved MDPSCs at the 7th passage of culture were stellate with short processes or spindle-shaped. The cell viability was more than 90% following thawing of the frozen cells. The doubling time was approximately 30 h as previously isolated from canine teeth transported by land within 1 h [9], suggesting that the transportation of the extracted teeth by air within 30 h did not affect the cell proliferation ability. The mRNA expression levels of *PTGES*, *COX-2*, *IL-6*, *TGF-β*, and *IDO-1* were similar in MDPSCs and MADSCs derived from the same individual dog (Table 5), suggesting similar immunomodulatory/immunosuppressive function of MDPSCs to MADSCs.

**Table 5:** Relative mRNA expression of immunomodulatory factors in MDPSCs compared with that in MADSCs

	MDPSC/MADSC
*PTGES*	2.6 ± 3.5
*COX-2*	0.9 ± 0.7
*IL-6*	1.3 ± 0.6
*TGF-β*	1.6 ± 0.7
*IDO-1*	1.5 ± 1.9

*MPDSC* mobilized dental pulp stem cell, *MADSC* mobilized adipose derived stem cell, PTGES prostaglandin E synthase, COX-2 cyclooxygenase-2, IL interleukin, TGF transforming growth factor, IDO-1 indoleamine 2,3-dioxygenase 1

### Safety of allogeneic transplantation

Toxicology assessment showed no adverse effects on appearance, clinical signs, food consumption, and body weight for 12 weeks after allogeneic first transplantation of the MDPSCs from four DLA-nonidentical donors as well as those from three DLA-identical and one almost DLA-identical donors. The blood test demonstrated no increase of white blood cell and platelet numbers (Table 6), demonstrating no alloreaction toward the transplanted cells. Serum and urine chemistry parameters showed values within normal ranges at 4 and 12 weeks after both first and second allogeneic transplantation (Table 6). Furthermore, there was also no evidence of toxicity or adverse events at 4 and 12 weeks after second DLA-nonidentical and DLA-identical transplantation of the same type of MDPSCs as in the first transplantation. No abnormalities were caused by the allogeneic transplantation in any organ or tissues assessed by histopathological examinations at 12 weeks after the second transplantation. These results demonstrate that DLA mismatched transplantation might be safe for pulp regeneration for 12 weeks in dogs not only the first time but also the second time.

**Table 6:** Safety evaluation by hematology and blood chemistry at 4 and 12 weeks after the first and the second allogeneic transplantation

Individual number	RBC (10^6^/μl)				WBC (10^3^/μl)				Platelet count (10^3^/μl)				Hematocrit (%)											
	First transplant		Second transplant		First transplant		Second transplant		First transplant		Second transplant		First transplant		Second transplant									
	4 weeks	12 weeks	4 weeks	12 weeks	4 weeks	12 weeks	4 weeks	12 weeks	4 weeks	12 weeks	4 weeks	12 weeks	4 weeks	12 weeks	4 weeks	12 weeks								
Hematology																								
13MW 302	6.33	6.79	6.65	6.73	12.78	12.94	11.91	13.65	286	316	281	259	42.5	46.1	44.2	44.4								
13FW 191	6.62	6.58	7	7.32	7.03	10.49	9.08	10.83	388	538	434	400	43.4	45.8	47.6	48.1								
13FW 283	6.22	6.4	6.41	7	12.36	13.4	16.78	14.14	400	439	360	334	43.8	46.6	46.5	48.4								
13FW 956	7.03	6.8	6.84	7.51	8.9	12.99	10.9	10.77	362	342	323	321	46.6	45.9	46	49.4								
13MW 1029	7.3	6.87	7.31	7.57	9.19	12.31	11.82	14.42	300	249	256	274	48.9	46	47.8	50.6								
13FW 300	6.79	6.75	7.29	6.9	8.79	8.08	7.86	8.52	387	313	274	317	44.4	45.4	47.3	44.4								
13FW 299	7.71	7	7.39	8.34	11.35	9.68	10.92	9.27	306	259	277	221	52.4	47.8	50.6	55.9								
13FW 1031	6.56	7.16	8.01	6.9	11.74	15.75	15.64	12.87	327	300	364	345	44.5	50	55.3	46.9								
13FW 926	7.82	8.26	8.42	7.49	10.18	9.6	11.05	11.32	369	338	347	413	56.9	58.3	58.7	53.3								
13MW 1122	7.07	6.94	6.91	7.11	8.37	8.8	7.83	8.41	285	278	265	271	49.5	48.8	47.8	49.2								
	AST (IU/L)				ALT (IU/L)				Albumin (g/dl)				Globulin (g/dl)				Total cholesterol (mg/dl)				Glucose (mg/dl)			
	First transplant		Second transplant		First transplant		Second transplant		First transplant	Second transplant			First transplant		Second transplant		First transplant	Second transplant			First transplant		Second transplant	
	4 weeks	12 weeks	4 weeks	12 weeks	4 weeks	12 weeks	4 weeks	12 weeks	4 weeks	12 weeks	4 weeks	12 weeks	4 weeks	12 weeks	4 weeks	12 weeks	4 weeks	12 weeks	4 weeks	12 weeks	4 weeks	12 weeks	4 weeks	12 weeks
Blood chemistry																								
13MW 302	28	32	29	31	36	35	35	31	3.1	3	3.1	3.2	2.3	2.3	2.1	2.3	175	168	174	178	90	92	92	97
13FW 191	21	28	28	29	41	42	45	43	3.3	3.2	3.1	3.4	2.3	2.9	2.8	2.7	252	169	167	191	98	86	95	95
13FW 283	26	31	26	30	34	43	36	40	3.1	3.1	3	3	2.2	2.4	2.6	2.7	160	134	155	137	90	82	89	87
13FW 956	21	26	23	23	33	35	34	42	3.5	3.4	3.3	3.6	2.7	2.7	2.6	2.9	173	159	157	170	84	87	84	80
13MW 1029	26	29	29	30	55	57	57	65	3.1	3	3.1	3.1	2.9	2.7	2.7	3	179	158	166	163	83	79	88	87
13FW 300	30	34	35	26	35	41	35	32	3.4	3.6	3.5	3.6	2.2	2.3	2.1	2.3	156	124	125	232	98	87	93	100
13FW 299	29	26	27	27	30	31	31	32	3.4	3.2	3.2	3.2	2.2	2.4	2.3	2.7	137	162	128	145	90	96	93	90
13FW 1031	18	24	24	18	37	39	37	41	3.1	3.3	3.5	3.3	2.3	2.5	2.6	2.4	237	185	261	232	98	97	93	97
13FW 926	29	28	41	22	50	56	54	157	3.6	3.6	3.7	3.4	2.8	3	2.8	3.1	172	176	173	256	86	79	85	92
13MW 1122	24	23	26	27	38	39	38	41	3.2	3.2	3.1	3.1	2.5	2.6	2.4	2.6	200	178	179	172	95	95	98	112

*RBC* erythrocyte count (red blood cells), *WBC* lymphocyte count (white blood cells), *AST* aspartate transminase, *ALT* alanine transminase

### Pulp regeneration after allogeneic transplantation

**Fig. 2:**
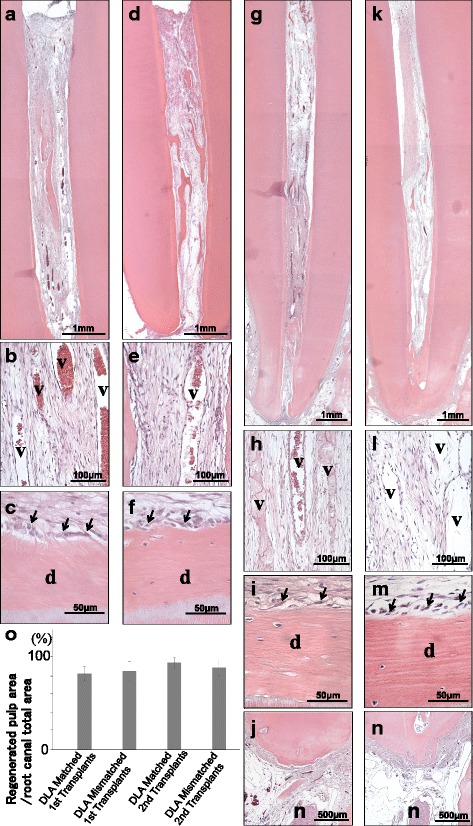
Total pulp regeneration 12 weeks after allogeneic transplantation of mobilized dental pulp stem cells (MDPSCs) in pulpectomized teeth in dogs. **a–f** First transplantation. **g–n** Second transplantation. **a–c, g–j** DLA matched transplant. **d–f, k–n** DLA mismatched transplant. **b, e, h, l** Newly formed blood vessels (v) in the regenerated tissue. **c, f, i, m** Odontoblast-like cells (arrows) lining along with the wall of the newly formed osteodentin/tubular dentin (d). **j, n** Periapical region of the transplanted teeth. Inferior alveolar nerve fiber bundle (n). Hematoxylin and eosin (HE) staining. **o** Morphometric statistical analysis. Ratio of newly regenerated area to root canal area. Data are mean ± standard deviation (*n* = 5). Tukey’s multiple comparison test method

**Fig. 3:**
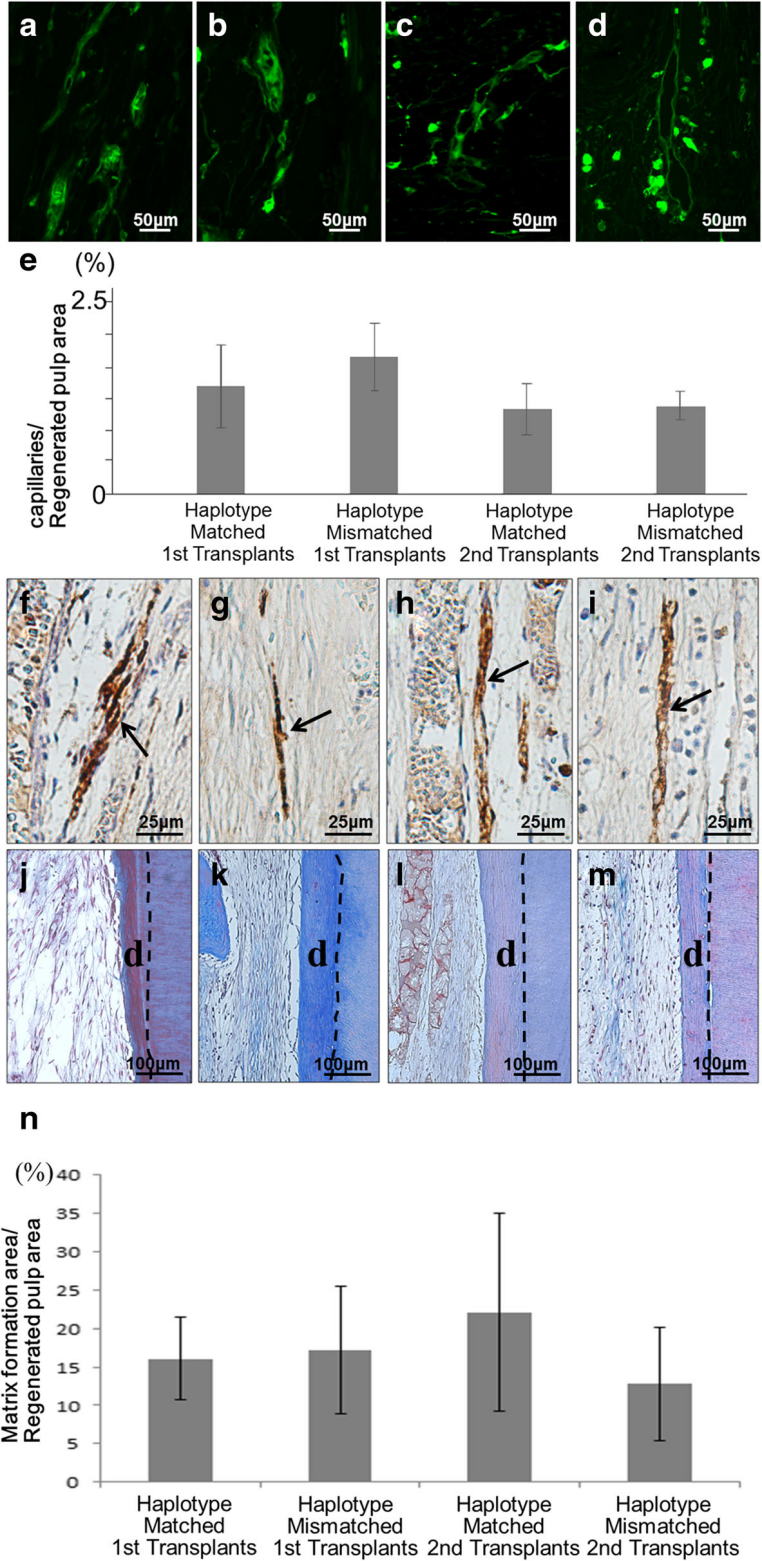
Histochemical analyses of regenerated pulp tissue. Immunostaining with (**a–d**) BS-1 lectin, (**f–i**) PGP 9.5 (arrows indicate neurite extension), and (**j–m**) Masson trichrome staining (hatched lines indicate newly formed osteo/tubular dentin (d)). **a, b, f, g, j, k** First transplantation. **c, d, h, i, l, m** Second transplantation. **a, c, f, h, j, l** DLA matched transplant. **b, d, g, i, k, m** DLA mismatched transplant. **e** Ratio of positively stained area by BS-1 lectin in a frame 310 μm × 240 μm in size. Data are mean ± standard deviation (*n* = 4). Tukey’s multiple comparison test method. **n** Ratio of Masson trichrome positively stained area to pulp regenerated area. Data are mean ± standard deviation (*n* = 3). Tukey’s multiple comparison test method

We next compared the regenerated tissue after DLA mismatched MDPSC transplantation with matched MDPSC transplantation into the pulpectomized root canal (Fig. 2). Pulp-like tissues with well-developed vasculature were observed at 12 weeks in both the allogeneic first transplants (Fig. 2a, b, d, e and Additional file 1: Figure S1). Similar pulp-like tissues in cell morphology, cell density, and architecture of the extracellular matrix with a few inflammatory cells were also regenerated at 12 weeks in both the DLA mismatched and matched second transplants (Fig. 2g, h, j, k). Odontoblast-like cells with a long process and osteodentinoblast-like cells were attached to the wall of newly formed osteo/tubular dentin (Fig. 2c, f, i, m) and neither inflammation nor internal/external resorption was detected (Fig. 2j, n). The regenerated tissue was filled in the root canal more than 80% to the dentin–enamel junction in all four transplants. The statistical analysis demonstrated no difference among the four transplants (Fig. 2o). Vascularization demonstrated by BS-1 lectin staining was also similar in density and orientation in the four groups of transplants (Fig. 3a–d), and there was no significant differences in the capillary density among them (Fig. 3e). Nerve fibers stained by PGP9.5 antibody were similarly observed, indicating similar reinnervation (Fig. 3f–i). Dentin-like mineralized tissue formation was similarly observed along the dentinal wall (Fig. 3j–m). There was no significant difference in the matrix formation among the four transplants by morphometric analysis of the Masson trichrome positively stained area (Fig. 3n). These results suggest no qualitative and quantitative differences in the regenerated tissue between the DLA mismatched and matched transplants and between first and second transplants.

## Discussion

The aim of the present investigation was to evaluate the safety and efficacy of allogeneic transplantation of DLA matched and mismatched MDPSCs in pulpectomized teeth with complete apical closure for pulp/dentin regeneration in dogs. A crucial challenge, however, is the limitation of genotyping major histocompatibility with relevance for humans using animal models in a preclinical study. The dog is a suitable animal model for pulp regenerative therapy, where the incisor tooth and its dental pulp tissue are similar in anatomy and developmental biology to humans [39]. Tissue regeneration in the dog may also be influenced by similar factors as in humans, including the immune system [40] and genetics [41]. Thus, dogs have served as an effective, directly translatable model for MSC transplantation [42, 43]. Major histocompatibility complex (MHC) genes in mammals include class I and class II genes that are highly polymorphic and their donor–recipient matching is important for cell transplantation [43]. The genes for the dog MHC or DLA have been defined as a sequence-based nomenclature [44, 45]. There are four complete class I genes: DLA-88, DLA-12, DLA-64, and DLA-79, in which DLA-88 is highly polymorphic (more than 72 alleles) and the others are less polymorphic. In the class II region, there are DLA-DQA1 (nine alleles), DLA-DQB1 (20 alleles), DLA-DRB1 (at least 24 alleles), and DLA-DRA (monomorphic) [43, 46]. In the present work, DLA genotyping and matching analysis were performed in 26 dogs by PCR for the four genes orthologous to the human genes including DLA-88, DLA-DQA, DLA-DQB, and DLA-DRB [47]. The results demonstrated eight dogs with four completely matched alleles (four homozygous allele profile), four dogs with three completely matched alleles (two homozygous and two heterozygous allele profile), and four dogs with three completely matched alleles (four heterozygous allele profile). The similarity of the allele profiles was not due to related siblings, and all of the MDPSCs were transplanted into unrelated recipients. Based on an analysis of canine DLA diversity, the three-locus DLA haplotype, DQA1 00101;DQB1 00201;DRB1 00102 represented 40.3% and DQA1 00101;DQB1 00201;DRB1 00101 represented 11.9% in the beagle [37], which is higher than the present rate: eight dogs out of 26 (30.7%) and only one dog out of 26 (3.8%), respectively, in the present study, suggesting the possibility of a distinct breed. The true extent of diversity of DLA genes in canines, especially of the class I gene DLA-88, however, is still unknown [43].

A variety of animal and human studies have demonstrated that stem cell-based therapy with allogeneic MSCs is a potential therapeutic option to regenerate damaged tissue/organ [10]. The low immunogenicity and immunomodulatory/immunosuppressive properties of allogeneic MSCs may contribute to a reduced immune response [48]. We have previously demonstrated lack of expression of MHC class II and costimulatory molecules, such as CD40, CD80 (B7-1), and CD86 (B7-2), although MHC-I is expressed in human MDPSCs [38]. We have also demonstrated that human and canine MDPSC conditioned medium inhibits allogeneic peripheral blood mononuclear cell (PBMC) proliferation and demonstrates a dose-dependent inhibition of PBMC immune responses in mixed lymphocyte reaction (MLR) assays [8, 38], confirming the work in DPSCs [49]. IFN-γ secreted by activated PBMCs induce the expression of soluble factors by DPSCs, which may play an important role in the immunosuppressive process [50]. Expression of PGE2, TGF-β, indolamine-2, 3-dioxygenase-1 (IDO1), IL-6, IL-10, and COX2 triggers the immunosuppressive activity of DPSCs [51–55]. The present study demonstrated that genes related to immunomodulation including *prostaglandin E synthase* (*PTGES*), *COX-2*, *IL-6*, *TGF-β*, and *IDO1* were similarly expressed in canine MDPSCs compared to canine mobilized adipose-derived MSCs. Fas ligand (FasL) associated with the immunoregulatory properties of DPSCs in the context of inducing T-cell apoptosis [56] was also expressed in MDPSCs (data not shown). The present results are consistent with the previous studies in human MSCs [57–59], demonstrating low immunogenicity of MDPSCs and potential to induce immune tolerance in the hosts.

Furthermore, MSCs can inhibit the immune response not only by suppressing T cells and by inducing Tregs but also by converting macrophages into a regulatory phenotype [60]. Polarization of macrophages toward the anti-inflammatory M2 phenotype has been reported after DPSC transplantation in diabetic peripheral nerves [61]. In addition, similar findings were seen by injection of conditioned medium from stem cells of human exfoliated deciduous teeth (SHED) into acute lung injury [62], autoimmune encephalomyelitis [63], and rheumatoid arthritis [64]. Further investigation is necessary to examine the shift in the macrophage phenotype from M1 to M2 in the regenerated pulp and periapical tissue after allogeneic DPSC transplantation.

MSCs express detectable levels of MHC class I and low levels of MHC class II to avoid recognition by a host immune system in allogeneic therapies [65, 66]. Several animal and clinical studies, however, have demonstrated that MSCs are weakly immunogenic in vivo in the case of transplantation across MHC class I barriers and that MSCs are rejected to induce chronic immune responses [31, 67–69]. Such responses could restrict the effectiveness of repeated transplantation of allogeneic MSCs [69]. Syngeneic and minor mismatched transplantation of synovial MSCs demonstrated more optimal meniscus regeneration compared with major mismatched transplantation in a meniscectomized model [70]. On the other hand, the correlation between the number of donor–host MHC mismatches and the efficacy of treatment was not detected in local injection in osteoarthritis and degenerative disc disease [71]. Furthermore, osteogenically differentiated MSCs are immunomodulatory and lack immunogenicity, demonstrating potential use in bone repair. However, maintenance of these properties in vivo is still open to question since immunogenic markers are upregulated after transplantation of the differentiated MSCs [72]. In the present study there was no correlation between the number of donor–host mismatches and efficacy, demonstrating the lack of immune response. This may be due to reduced host immune responses by the transplanted MDPSCs and effective confinement of these cells into the root canal of the tooth. Another possibility is that the transplanted MDPSCs are not differentiated into any host cells in the regenerated pulp tissue. Our previous study demonstrated that injected DiI-labeled autologous MDPSCs did not differentiate into host cells and induced pulp regeneration by secreting trophic factors to elicit migration and proliferation and inhibit apoptosis of endogenous MSCs [8]. We further demonstrated that transplanted porcine pulp MSCs were not directly incorporated into endothelial cells, neuronal cells, or host pulp cells in mouse ectopic tooth transplantation models [6].

Although MSCs are known to be immunoprivileged, repeated transplantation of mismatched MSCs has been reported to lead to alloimmunization and subsequent refractoriness in mice [67, 73]. Multiple intravenous injections of allogeneic MSCs are well tolerated in healthy horses, indicating no clinical signs of organ toxicity and systemic inflammatory response. A mild alloantigen-induced cytotoxic response, however, is suggested by an increased numbers of circulating CD8^+^ T cells [32]. Dual injection of allogeneic MSCs into joint and articular cartilage induces an adverse clinical response, suggesting immune recognition of allogeneic MSCs after the second infection [74, 75]. Allogeneic MSCs are weakly immunogenic when transplanted across MHC boundaries in rhesus monkeys, indicating negative impact by dual transplantation [76], while repeated intravenous injection of human umbilical cord blood-derived MSCs has low immunogenicity and no adverse events detected in mice [34] and humans [77]. Furthermore, there are no toxicological abnormalities and no obvious pathological changes although CD3^+^ and IL-6 levels are significantly increased after repeated intravenous injection of monkey umbilical cord MSCs [78]. In the present study, there were no toxicological abnormalities and no significant difference in tissue volume of regenerated dental pulp and inflammatory cell infiltration between the first and second transplants for both DLA matched and mismatched MDPSCs. This result demonstrates that MDPSCs are immunologically safe for use in allogeneic applications.

## Conclusion

In this preclinical study, the safety of allogeneic mismatched MDPSC transplantation in pulpectomized teeth was demonstrated. Regenerated pulp tissues including neovascularization and neuronal extension were similar in the DLA mismatched transplants compared to the DLA matched transplants even after dual transplantation of MDPSCs, suggesting efficacy for total pulp regeneration.

## Additional file

Additional file 1: **Figure S1.** Histochemical analyses of normal pulp tissue. Immunostaining with (**A**) BS-1 lectin and (**B**) PGP 9.5. Neurite extension (arrow). (PDF 155 kb)

## Abbreviations

G-CSF: Granulocyte colony-stimulating factor
MDPSC: Mobilized dental pulp stem cell
