# Supplementary figures and images for: Allogeneic transplantation of mobilized dental pulp stem cells with the mismatched dog leukocyte antigen type is safe and efficacious for total pulp regeneration
Source: Stem Cell Res Ther. 2018 Apr 27;9:116. doi: 10.1186/s13287-018-0855-8 (PMC5921747; doi:10.1186/s13287-018-0855-8)

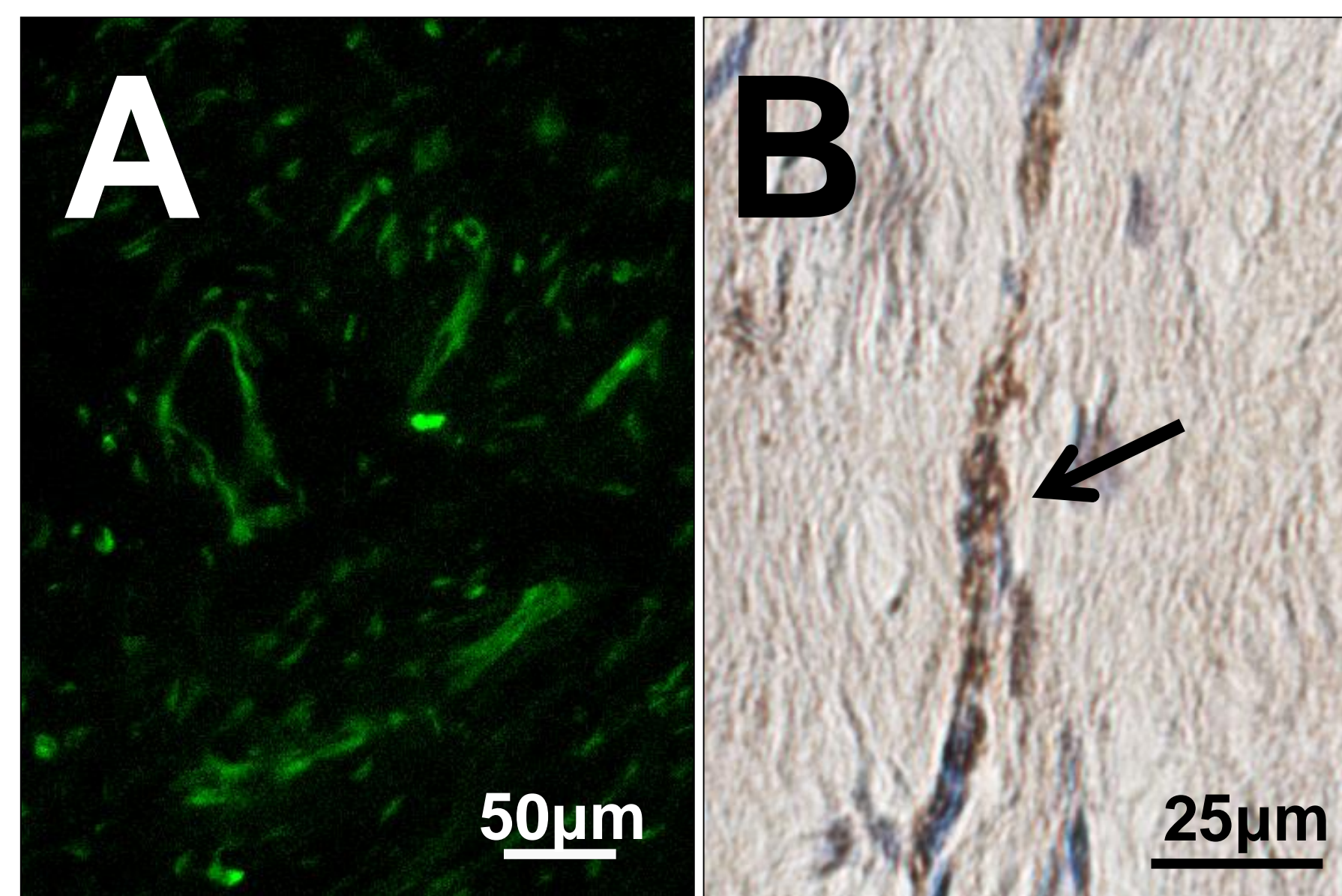

**Figure S1**

Supplement: Supplementary file 1 — Figure S1. Histochemical analyses of normal pulp tissue. Immunostaining with (A) BS-1 lectin and (B) PGP 9.5. Neurite extension (arrow). (PDF 155 kb) [file 13287_2018_855_MOESM1_ESM.pdf]
